# An Artificial Intelligent Risk Classification Method of High Myopia Based on Fundus Images

**DOI:** 10.3390/jcm10194488

**Published:** 2021-09-29

**Authors:** Cheng Wan, Han Li, Guo-Fan Cao, Qin Jiang, Wei-Hua Yang

**Affiliations:** 1 College of Electronic and Information Engineering, Nanjing University of Aeronautics and Astronautics, Nanjing 211100, China; wanch@nuaa.edu.cn (C.W.); lhlyx_mail@163.com (H.L.); 2 The Laboratory of Artificial Intelligence and Bigdata in Ophthalmology, The Affiliated Eye Hospital of Nanjing Medical University, Nanjing 210029, China; caoguofan587@163.com (G.-F.C.); jqin710@vip.sina.com (Q.J.)

**Keywords:** high myopia, fundus images, computer-aided diagnosis, risk classification

## Abstract

High myopia is a global ocular disease and one of the most common causes of blindness. Fundus images can be obtained in a noninvasive manner and can be used to monitor and follow up on many fundus diseases, such as high myopia. In this paper, we proposed a computer-aided diagnosis algorithm using deep convolutional neural networks (DCNNs) to grade the risk of high myopia. The input images were automatically classified into three categories: normal fundus images were labeled class 0, low-risk high-myopia images were labeled class 1, and high-risk high-myopia images were labeled class 2. We conducted model training on 758 clinical fundus images collected locally, and the average accuracy reached 98.15% according to the results of fivefold cross-validation. An additional 100 fundus images were used to evaluate the performance of DCNNs, with ophthalmologists performing external validation. The experimental results showed that DCNNs outperformed human experts with an area under the curve (AUC) of 0.9968 for the recognition of low-risk high myopia and 0.9964 for the recognition of high-risk high myopia. In this study, we were able to accurately and automatically perform high myopia classification solely using fundus images. This has great practical significance in terms of improving early diagnosis, prevention, and treatment in clinical practice.

## 1. Introduction

High myopia is a global ocular disease. In recent years, its global incidence has increased significantly. The increasing number of people suffering from myopia and high myopia is not only a serious human health problem, but also a public management problem that affects social development. The cost of preventing and treating eye complications and vision loss in the nearly 1 billion people with high myopia is extremely high. Studies [1,2] have shown that, on a global scale, the loss of productivity due to uncorrected vision impairment is approximately USD 121.4 billion, while the cost of facilities and personnel required to establish refractive treatment services is as high as USD 20 billion [2]. The incidence of myopia in East Asia is significantly higher than that in Western countries, especially among young people in East Asia [1]. High-myopia fundus disease has become the second leading cause of blindness in China, and the trend tends towards a younger population [3]. Studies [1,2] have shown that in 2000, 2010, 2020, 2030, 2040, and 2050, the global prevalence of myopia for all age groups was/will be 22.9%, 28.3%, 34.0%, 39.9%, 45.2%, and 49.8%, respectively. Additionally, the prevalence of high myopia was/will be 2.7%, 4.0%, 5.2%, 6.1%, 7.7%, and 9.8%, respectively. It is estimated that by 2050, 4.758 billion people will suffer from myopia, and of these, 938 million will suffer from high myopia; this represents almost 50% and 10% of the world’s total population, respectively [1]. According to the statistics, the overall incidence of myopia in children and adolescents in China is 53.6%, and the overall incidence of myopia in college students exceeds 90%. Among them, the prevalence of high myopia ranges from 6.69% to 38.4% [3]. Therefore, prevention and control of high myopia is a matter of great importance for society as a whole.

The difference between high myopia and low–medium myopia is that in high myopia, the refractive power in diopters is very high, usually characterized by a refractive error of ≤−6.00 D. Moreover, it is mainly characterized by axial elongation. As myopia deepens and the axis of the eye grows longer, the visible retinal choroidal disease at the fundus becomes more severe, causing a range of serious complications, most of which lead to blindness. For this reason, high myopia-related fundus disease is a common cause of blindness worldwide and the second leading cause of blindness in China [3]. Although the terms “high myopia” and “pathological myopia” are often used interchangeably in daily life, they do not actually refer to the same disease. According to the consensus issued by the Optometry Group of the Ophthalmology Branch of the Chinese Medical Association in 2017 [3], high myopia can be divided into simple high myopia and pathological myopia. Although simple high myopia features high myopic diopters and has symptoms such as decreased vision, asthenopia, floaters, etc., it is not accompanied by serious fundus damage. However, the symptoms of pathological myopia include more serious impairment of visual function, such as occlusion, deformation, and visual ghosting, in addition to decreased vision, and the resulting fundus diseases are permanent and irreversible [3]. In addition, the degree of simple high myopia tends to be stable in adulthood, while the degree of pathological myopia deepens continuously as the course of disease progresses, accompanying the patient for life [4]. The potential risks of these two diseases are completely different. There is an urgent need for a standard risk-grading system with consistent nomenclature with which to classify different levels of risk for high myopia. This can then be used in different studies to assess the therapeutic utility. Therefore, we proposed an intelligent classification method for high myopia images according to the degree of potential risk. The specific classification rules are described in the Data Collection section below.

The traditional detection of high myopia mainly relies on artificial auxiliary methods such as diopter detection, eye axis measurement, and fundus color photography. However, manual testing and analysis rely on ophthalmologists, which is time-consuming and labor-intensive [4]. In addition, the shortage of ophthalmologists and medical equipment in areas with relatively poor medical resources leads to patients missing the optimal treatment period. For this reason, it is important to develop intelligent eye disease diagnostic methods based on fundus images. Clinically, for common fundus diseases such as diabetic retinopathy, macular degeneration, and glaucoma, fundus images have been widely used in disease diagnosis because they are noncontact, nondestructive, low-cost, easy to obtain, and easy to process.

In recent years, with the rapid development of the artificial intelligence technology and deep learning methods, many researchers have applied them to various image processing problems. New techniques and methods that analyze fundus images for high myopia have been continuously emerging [5,6,7,8,9,10]. These methods use computer-aided technologies to automatically analyze and diagnose lesions associated with high myopia in the absence of experienced ophthalmologists and professional optometry instruments. For example, Liu et al. [5] first proposed a system named PAMELA to detect pathological myopia. They used the support vector machine (SVM) approach in the machine learning technology to extract texture features in fundus images to diagnose pathological myopia. However, compared with the relatively simple SVM, deep learning methods can extract more abstract high-dimensional features, thus greatly improving recognition accuracy. Currently, most image classification methods are based on the convolutional neural network (CNN) model in deep learning. Varadarajan et al. [6], a research team from Google, introduced an attention mechanism into a CNN and trained a deep learning model to predict the refractive error. Through an attention heat map, it was shown that the fovea region of the retina has the largest contribution to the prediction. Dai et al. [7] designed a CNN model with two branches. One branch was used to distinguish between normal and abnormal fundus images, and the other branch was used to distinguish between simple high myopia and pathological myopia images. However, this model required two-step judgment, which has a lower prediction speed than the end-to-end direct classification method. The first task of the 2019 PALM Color Fundus Photographic Pathological Myopia Challenge [8] was the qualitative classification of pathological myopia. Participating teams used different CNN models [9,10,11] to predict the risk of pathological myopia using fundus images. However, these classifications only distinguished pathological myopia from nonpathological myopia, and certain fundus images classified as nonpathological myopia still have a certain high myopia risk (such as “simple high myopia” defined in [3]).

## 2. Materials and Methods

### 2.1. Data Collection

We referred to the rules of the International Photographic Classification and Grading System for Myopic Maculopathy [12]. Fundus images were classified into three categories according to the risk of disease: normal fundus, low-risk high myopia, and high-risk high myopia. Specifically, the label of normal fundus was class 0, and there are no significant lesions in this category. Low-risk high myopia was labeled class 1 based on the presence of tessellated fundus. High-risk high myopia was labeled class 2 due to the presence of more severe lesions in the fundus. These severe lesions include diffuse chorioretinal atrophy, patchy chorioretinal atrophy, and macular atrophy. Additional features such as lacquer cracks, choroidal neovascularization, and Fuchs spot were considered to be plus signs that did not fit into any particular category and could develop from or occur in any category.

The dataset used in this study was provided by the Affiliated Eye Hospital of Nanjing Medical University. All the images were obtained from multiple models of nonmydriatic fundus cameras, and the resolution of each image ranged from 512 × 512 to 2584 × 2000. There were no restrictions on the age or gender of the patients represented by the images. Our study followed the principles of the Declaration of Helsinki. The collected images were all anonymized, i.e., all the patient-related personal information was removed to avoid infringing on patient privacy; thus, there were no relevant patient statistics. The true label of each fundus image was decided upon by two ophthalmologists using the double-blind approach. Two identical diagnoses from the physicians formed the final result. When the two physicians provided different diagnoses, the judgment of an additional expert ophthalmologist was used as the final result.

The dataset used in this study included 858 color fundus images of patients of all ages. In order to exclude subjective factors, we used a random number seed to randomly divide all the fundus images into dataset A and dataset B. Dataset A was used to train the deep convolutional neural networks (DCNNs), and dataset B was the external validation dataset, which was used to compare the diagnostic performance of the intelligent model with that of the human expert. The intuitive distribution of data categories is shown in Table 1.

### 2.2. Model Development

**Figure 1:**
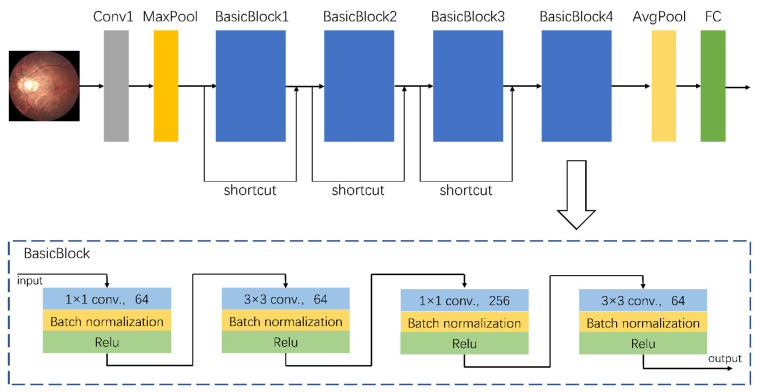
The architecture of the proposed deep convolutional neural networks. The grey block represents the input convolutional layer. The yellow blocks represent the maximum pooling layer or the average pooling layer. The blue blocks are four convolutional basic blocks whose detailed structure is shown at the bottom of the figure. The green block is the final full connected layer used for classification.

A total of 758 fundus images from dataset A were used to train the assisted diagnostic model. The trained DCNNs model can be used to efficiently obtain diagnostic results, i.e., for an input fundus image, the model automatically outputs the category to which the image belongs, namely the risk level of high myopia as predicted by the model. As the clinical data were collected from different camera instruments, the image resolution varied. Therefore, we first adjusted the size of all images to 224 × 224 for normalization, and carried out a series of preprocessing operations to enhance feature expression. These preprocessing operations mainly consisted of the following steps: quality assessment, contrast enhancement, image denoising, mean normalization, and variance normalization. Thereafter, we used the designed DCNNs for feature extraction and classification. The architecture of our proposed DCNNs is shown in Figure 1. Its basic network structure includes a convolution layer, a maximum/average pooling layer, a batch normalization layer, an activation layer, and a fully connected layer. Because the sample size of dataset A (which was used for training) was relatively small, there was a high risk of overfitting when the network model had too many parameters or too many layers. That is to say that when the accuracy of a network model on the training set is very high but the accuracy on a new dataset that has not been seen is very low, it does not have a powerful generalization ability. Therefore, the proposed DCNNs are mainly composed of a head convolution layer Conv1 and four continuous convolution modules named BasicBlocks. There are additional shortcut connections between the four convolution modules. Adding a shortcut connection is equivalent to adding all the information of the image of the previous layer in each block. To some extent, more original information is retained, and the possibility of a vanishing gradient problem in back propagation can be reduced. The final output of the fully connected layer was changed to three categories to accommodate the risk classification task of this study.

Experimental hardware: the CPU used was 3.60 GHz Intel(R) Core (TM) i7-7700 (Intel, Santa Clara, CA, USA); the GPU was NVIDIA GeForce RTX 2080-Ti (Micro-Star, Xinbei, China) with 16 GB of memory. Experimental software: the operating system used was Windows 7 × 64 (Windows 7, Microsoft, Redmond, WA, USA). The DCNNs model was built based on Pytorch (https://pytorch.org/, accessed on 17 July 2021) with Python (Python 3.6.5, Python Software Foundation, Delaware, DE, USA). In the training process, the limited amount of training data increased the probability of an overfitting problem. In order to prevent overfitting and enhance the robustness of the model, we augmented the training data in many different ways, including random horizontal and vertical flips, rotating in random directions, and modifying the brightness, contrast, and saturation to cause color disturbances, etc. A total of 758 images from dataset A were augmented, and the number of samples after augmentation was five times the original, that is, a total of 3790 samples were utilized in the training process. The training process used fivefold cross-validation, with 80% of the samples used for training and 20% used for validation in each iteration. Furthermore, we adopted the label-smoothing regularization [13] and dropout [14] methods to further prevent overfitting. The total number of epochs in the training process was set to 100, and the initial learning rate was 0.000002. The learning rate was updated by adopting a strategy combining warm-up [13] and cosine annealing. The optimizer was RAdam [15], and the weight decay was set to 0.001.

### 2.3. Statistical Analysis

**Figure 2:**
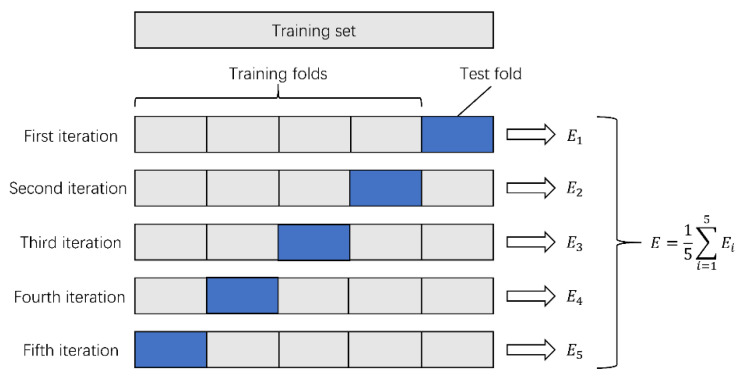
The process of fivefold cross-validation.

The collected data were randomly divided into two datasets: dataset A and dataset B. Model training was carried out on dataset A (a total of 758 images). Since the number of collected fundus images was relatively small, we needed to make full use of the data information to train DCNNs. Therefore, we adopted fivefold cross-validation in the training process. The specific steps were as described further. Dataset A was randomly divided into five parts. Each time, four parts were selected as the training set, and the remaining part was used as the test set. The cross-validation was repeated five times, and the average accuracy after the five cross-validations was taken as the final evaluation result of the DCNNs. The variance was reduced by averaging the results of the five different training groups. This way, the performance of the model was less sensitive to the division of data, and overfitting was reduced. On the other hand, cross-validation went through more iterations and thus avoided underfitting to some extent. The process of fivefold cross-validation is shown in Figure 2. This can effectively avoid the occurrence of overfitting and underfitting problems.

The 100 fundus images in dataset B were not used in the training process; instead, they were used as an additional test set for external validation. The 100 images were randomly selected by a computer program without any subjective bias, and the sample size of this study was calculated based on the sample size of previous studies. In order to evaluate the diagnostic level of our proposed algorithm in the real world, we compared the DCNNs model with another human expert. The human expert that participated in the external validation was a Chinese practicing physician who specialized in the clinical diagnosis and treatment of ophthalmic diseases. It should be noted that this human expert classified the cases independently and did not overlap with the group of ophthalmologists that annotated the dataset during the data collection process. To reduce the risk of prejudice caused by prior knowledge, the human expert was only allowed to observe the fundus lesions to determine the risk level of high myopia, without knowing the label information of the images or the patients’ medical history. The label of each fundus image was judged as “normal” (class 0), “low-risk high myopia” (class 1), or “high-risk high myopia” (class 2), which was consistent with the classification standard of the DCNNs model. Finally, we recorded the diagnosis results of the human expert and compared them with the results of the DCNNs model.

We used a confusion matrix and receiver operating characteristic (ROC) curves to evaluate the classification performance. The confusion matrix placed the predicted labels and the true labels in the same table by category. In this table, we could clearly see the number of correct identifications and misidentifications in each category. Moreover, the area under the curve (AUC) value calculated using ROC curves could be used to evaluate the performance of a classifier. The closer the AUC is to 1.0, the better the classification effect. The number of true-positive (TP), true-negative (TN), false-positive (FP), and false-negative (FN) samples from each category was calculated using the confusion matrix, and accuracy, sensitivity, specificity, positive predicted value (PPV), and negative predicted value (NPV) could be easily calculated.

This was a multiclass (class 0, 1, 2) classification task. However, most of the aforementioned evaluation indicators are applicable to binary classification with only positive and negative classes. Therefore, we used two methods to evaluate the results of the multiclass classification task. One of the methods involved transforming the multiclassification problem into multiple separate binary classification problems, i.e., for the identification of low-risk high myopia, we only regarded the images labeled class 1 as positive samples; other images (class 0 and class 2) were regarded as negative samples. Similarly, for the identification of high-risk high myopia, only images labeled class 2 were treated as positive samples, while other images (class 0 and class 1) were treated as negative samples. Another method involved using multiclassification indicators directly defined by the kappa score and the Jaccard score to assess the overall performance. The calculation of the kappa score was based on the confusion matrix; its definition is as follows:

(1) $$kappa=\frac{p_{o}-p_{e}}{1-p_{e}}$$

where $p_{o}$ represents the total classification accuracy, and the representation of $p_{e}$ is:

(2) $$p_{e}=\frac{a_{1}\times b_{1}+a_{2}\times b_{2}+\ldots +a_{c}\times b_{c}}{n\times n}$$

where $a_{i}$ is the number of real samples of class i; $a_{i}$ is the number of predicted samples of class i; and n is the total number of samples.

The Jaccard score measures the similarity between two sets of A and B as follows:

(3) $$\mathrm{Jaccard}\left(A,B\right)=\frac{\left|A\cap^{\;}B\right|}{\left|A\cup^{\;}B\right|}=\frac{\left|A\cap^{\;}B\right|}{\left|A\right|+\left|B\right|-\left|A\cap^{\;}B\right|}$$

where the sets A and B represent the true label set and the predicted label set of all the samples, respectively.

### 2.4. Diagnosis Visualization

In order to more intuitively analyze the influence of each area in a fundus image on the classification results, analyze the causes of wrong classification, and reasonably explain certain seemingly unreasonable results output by the model, we used gradient weighted class activation mapping (Grad-CAM++) [16] to perform visual analysis of the DCNNs.

Class activation mapping (CAM) is a visual analysis method that expresses the importance of each pixel to the image classification in the form of a heat map by performing a weighted summation on the feature maps corresponding to the network weights. However, CAM needs to modify the network structure and retrain the model. Considering the simplicity of the practical application, we used Grad-CAM++ to conduct the visual analysis in this study. Grad-CAM++ does not need to modify the network structure, and the characteristics learned by the model can be intuitively displayed without reducing the classification accuracy, which makes the model more transparent and interpretable. Specifically, Grad-CAM++ removes the fully connected layer in DCNNs and uses the output of the second-to-last convolutional layer to complete the visualization. It can intuitively display the important areas that are helpful for classification. The more important areas are represented by the warmer colors in the heat map.

## 3. Results

The model training process was carried out on dataset A (which had 758 images, including 233 normal images, 339 low-risk high myopia images, and 168 high-risk high myopia images) The advantage of cross-validation is that the randomly generated subsamples are repeatedly used for training and validation at the same time, and the results are validated once each time. Thus, the accuracy of the algorithm can be estimated more accurately, and a more reliable and robust model can be obtained. The detailed quantitative results of our proposed DCNNs are shown in Table 2.

**Figure 3:**
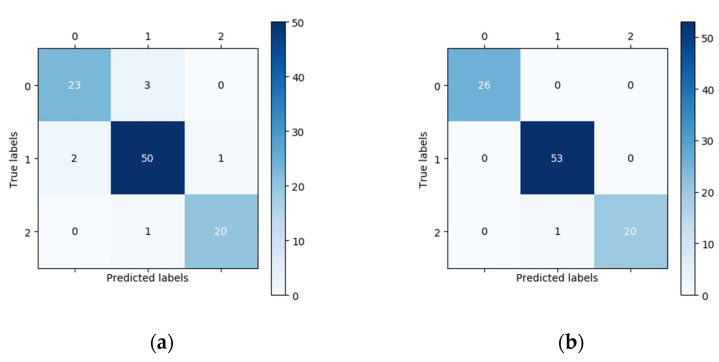
Comparison of the classification performance of the DCNNs and the human expert in the external validation of dataset B. The results are shown as a confusion matrix in which the vertical axis represents the true labels and the horizontal axis represents the predicted labels. (**a**) The confusion matrix of the human expert. (**b**) The confusion matrix of the DCNNs.

In this study, 100 fundus images (26 normal images, 53 low-risk high myopia images, and 21 high-risk high myopia images) were randomly chosen for external validation. The vertical axis of the confusion matrix in Figure 3 is the true label, while the horizontal axis is the predicted label. In the confusion matrix in Figure 3, we can see that the number of correctly classified images located on the main diagonal in the confusion matrix of the DCNNs is significantly higher than that corresponding to the confusion matrix of the human expert. For the classification of the 26 normal fundus images, the results of the DCNNs were all correct, whereas the human expert only correctly classified 23 images and wrongly classified three images as low-risk high myopia. Using the confusion matrix, we were able to easily calculate the sensitivity, specificity, and other indicators. The performances of the DCNNs were compared with those of the human expert using descriptive statistics. All of the statistical tests in our study were two-sided, and a *p*-value less than 0.05 was considered significant. In terms of the overall classification accuracy, the correct and incorrect samples in the human expert diagnosis accounted for 93% and 7%, respectively. After using the DCNNs, the correct rate of diagnosis increased to 99% and the incorrect rate decreased to 1%. Seven samples that were incorrectly diagnosed by the human expert were correctly diagnosed using the DCNNs, and one sample that was correctly diagnosed by the human expert was incorrectly diagnosed after using DCNNs. McNemar’s test showed that there was a statistically significant difference in the proportion of correct diagnoses before and after the DCNNs were used.

**Figure 4:**
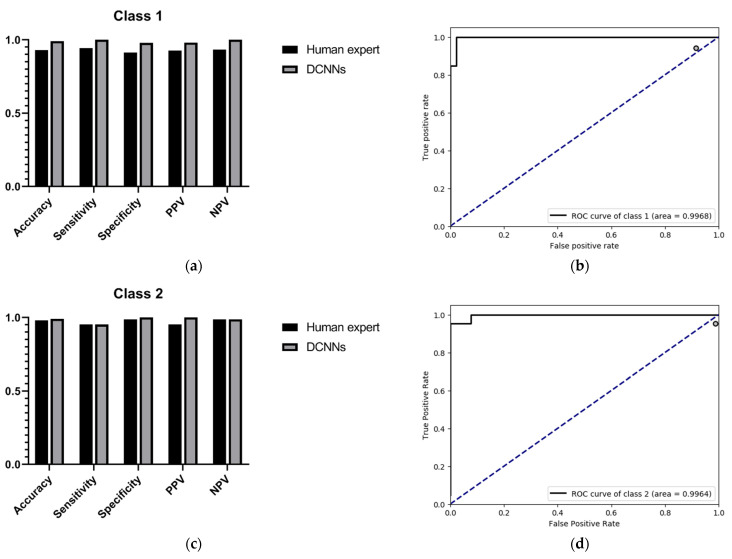
Evaluation results in the external validation dataset B. (**a**) Accuracy, sensitivity, specificity, PPV, NPV of the recognition of class 1; (**b**) ROC curves and AUC value of the recognition of class 1; (**c**) accuracy, sensitivity, specificity, PPV, NPV of the recognition of class 2; (**d**) ROC curves and AUC value of the recognition of Class 2.

Since the purpose of this study was to correctly identify high myopia images and decide upon risk classification, we only show the performance comparison between the DCNNs and the human expert as regards predicting low-risk high myopia (class 1) and high-risk high myopia (class 2). The specific results are shown in Table 3 and Table 4. For the recognition of low-risk high myopia images (class 1), the DCNNs model had a superior performance in most indicators; the sensitivity and the NPV of the DCNNs were both 100%, while the sensitivity and the NPV of the human expert were 94.34% and 93.48%, respectively. For the recognition of high-risk high myopia images (class 2), the DCNNs model had a comparable sensitivity of 97.83% and an NPV of 98.75%, which is equivalent to those of the human expert. However, the DCNNs performed very well in specificity and PPV, both of which were 100%. It can also be seen from the ROC curves in Figure 4 that our proposed DCNNs had an AUC of 0.9968 for the recognition of low-risk high myopia and 0.9964 for the recognition of high-risk high myopia. Moreover, the sensitivity–specificity points corresponding to the human expert were all below the black curves corresponding to the DCNNs, which indicates that the DCNNs achieved a superior performance as compared to the human expert.

In addition to transforming the multiclassification problem into several separate binary classification problems, we also directly introduced two multiclassification indicators to assess the overall performance: the kappa score and the Jaccard score. The kappa score is a method used in statistics to evaluate consistency. The higher the kappa value, the higher the classification accuracy of the algorithm. The Jaccard score is used to compare the similarities and differences between finite sample sets. In this study, we compared the similarities between the ground-truth set of the validation images and the corresponding prediction set. The larger the Jaccard score, the higher the sample similarity. We compared the performance of the DCNNs with that of the human expert in Table 5. The experimental results showed that both the kappa and Jaccard scores of the DCNNs were significantly better than those of the human expert.

**Figure 5:**
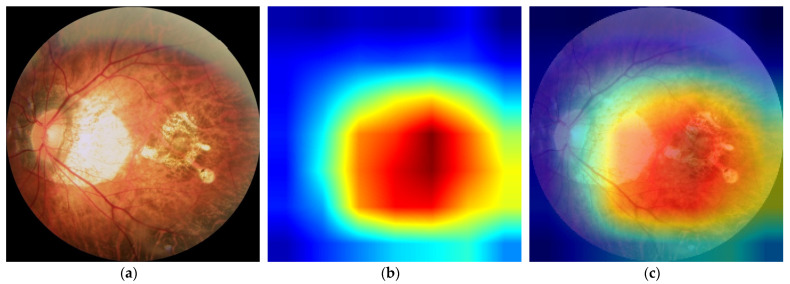
Visualization-based diagnosis by the DCNNs. (**a**) Original image. (**b**) Heatmap. (**c**) Grad-CAM++. Heatmaps and Grad-CAM++ showed that the DCNNs model focused on the macular area and the area of choroidal atrophy rather than the background.

As shown in Figure 5, we used gradient activation heatmap Grad-CAM++ to analyze the fundus image area. From left to right, we can see the original fundus image, the corresponding heatmap, and the corresponding gradient activation heatmap (Grad-CAM++) superimposed on the original image area. The warmer the color in the heatmap, the greater the influence on the classification prediction result. Figure 5a shows a high-risk high myopia fundus image with severe lesions, including diffuse chorioretinal atrophy, patchy chorioretinal atrophy, and macular atrophy. We can see that the DCNNs mainly focused on the macular area and the area of choroidal atrophy, rather than the background or other parts in the fundus.

## 4. Discussion

This study shows that the DCNNs model can be trained to detect high myopia and identify subtle lesion features in retinal fundus images in order to diagnose the risk of high myopia. Traditionally, myopia is diagnosed and classified through functional examinations (e.g., eye tests, optometry, microscopic field of vision tests, electrophysiological assessments) [17] or ocular coherence tomography (OCT), retinal autofluorescence (AF), fluorescein angiography (FA), or indocyanine green angiography (ICGA), which analyze the morphological changes of suspected pathological myopia [17]. Experimental results show that the DCNNs model is superior to or equivalent to a human expert in terms of accuracy, sensitivity, specificity, PPV, NPV, and other indicators. As compared with time-consuming and labor-intensive manual assisted examinations, the proposed method can reduce costs and improve diagnostic efficiency and accuracy. In addition, regarding the risk classification of high myopia, ophthalmologists are susceptible to subjective cognition and empirical impressions, which can lead to great differences in classification. The DCNNs model only performs feature extraction and prediction from the image level. It only needs to specify a standardized grading standard when labeling the training data, and the DCNNs model can give a prediction result that meets the grading standard.

As can be seen from Table 1, the fivefold training losses were very small, and the training accuracy remained at 100%, which indicates that the network model could adequately fit the training data. As a result of the relatively small size of the training dataset, we must pay attention to the possible overfitting problem. The average validation accuracy of the DCNNs was 98.15% according to the results of fivefold cross-validation, indicating that the model has good robustness. In the external validation set, we performed a classification performance comparison between the DCNNs and the human expert, which highlights the progress and practical significance of our method. In the randomly acquired external validation set, low-risk high myopia (class 1) accounted for more than half of the images. The unbalanced category distribution greatly increased the difficulty of classification. The confusion matrices in Figure 3 show that the human expert misdiagnosed three normal images as low-risk high myopia, missed two low-risk myopia cases and confused two images of low-risk and high-risk high myopia. The DCNNs correctly identified almost all the images, except for one that was considered to be a high-risk case image when in fact it was low-risk. On the one hand, when transforming the multiclassification problem into several separate binary classification problems, whether using the quantitative indicators such as accuracy, specificity, sensitivity, NPV, and PPV or the intuitive comparison of ROC curves, the DCNNs achieved a better or equal classification level as compared with the human expert. On the other hand, when directly using the kappa and Jaccard scores for the overall assessment of multiclassification problems, the scores of the DCNNs were both above 0.98, i.e., significantly better than those of the human expert. Since there are no clinical “gold standards” for the risk classification of high myopia, different ophthalmologists may have very different judgments due to the influence of subjective cognition and experience. As compared with ophthalmologists, DCNNs can always give prediction results that meet the given grading standards, which are not susceptible to other subjective factors and have higher generalization ability. The visualization results in Figure 5 show that the DCNNs model is hardly affected by the background when performing classification and mainly focuses on the macular area and the surrounding area of the optic disc in the fundus image. These areas are also the focus of the pathological characteristics of the fundus with high myopia, indicating that the classification of DCNNs is interpretable.

Most computer-aided algorithms for detecting high myopia are designed for binary classification. There are algorithms that only distinguish between normal and high myopia, or algorithms that only distinguish between normal and pathological myopia. However, it is also of great practical significance to classify the risk grade of high myopia. High myopia has obvious clinical manifestations, and physicians can easily judge whether there is high myopia based on the level of vision. However, assessing the severity of high myopia is a more challenging task. Even experienced ophthalmologists need to take great care in their observations in order to make a diagnosis. As compared with the model proposed by Dai [7] that judges the severity of high myopia according to two branches, our method achieves direct end-to-end classification and has significant advantages related to prediction speed. The real-world clinical value and utility of this study are as follows: the proposed intelligent classification model can be used in community hospitals and eye health institutions, such as physical examination institutions, and optician shops. When community doctors or staff at an institution collect fundus images, they will be able to predict the risk of high myopia and make recommendations to the patient, including certain recommendations regarding referrals.

Our study has several advantages. First, our DCNNs model can automatically determine the risk classification of high myopia. Using only retinal fundus images, it achieved a better diagnostic accuracy than a human expert. Moreover, it can automatically process data without manual assistance and with higher execution efficiency. Second, our model focuses on extracting morphological features from the image and making a prediction. This is not easily affected by subjective cognition and experience. No matter what grading standard is given, the prediction results of the network model are always consistent with the grading standard used to label the training data. Third, we applied the deep learning technology for the first time to classify the risk of high myopia. Not only did we automatically detect high myopia in the fundus images, but we also predicted its severity, which has a great clinical significance in real-life situations.

This study has several limitations. First, the distribution of sample categories was uneven. Low-risk high myopia images accounted for the largest proportion in the dataset, but normal fundus images would be the most common in real-life screening scenarios. In the confusion matrix, it can be seen that the diagnosis accuracy of the human expert for low-risk high myopia was lower than for high-risk high myopia. In future research, a larger sample size, a more balanced sample distribution, and more increased training data are required in order to improve the classification accuracy of our model. Second, the application of the DCNNs model needs to be evaluated using different ethnic groups in order to verify the robustness of our model in the risk classification of high myopia. The research objects in this paper were all Asian, and, due to the limited dataset, no validation experiments on other races were conducted. Third, as a result of differences in the symptoms of high myopia in adolescents and elderly people, in the future, age, gender, family genetic history, and other factors should be considered when building intelligent models in order to improve the algorithm performance in the high myopia risk classification task.

## 5. Conclusions

Herein, we propose a method for risk classification of high myopia based on a DCNNs deep learning model which can automatically classify fundus images into three categories: normal, low-risk high myopia, and high-risk high myopia. The proposed method achieved a diagnosis accuracy superior to that of a human expert. Moreover, we applied the deep learning technology to the risk classification of high myopia for the first time. We believe that this method can be widely used in myopia screening and could be of great significance to clinical practice. This approach allows for the efficient and effective prediction of high myopia severity, which is conductive to assessing the potential risk and providing timely treatment.

## Figures and Tables

**Table 1:** Distribution information of datasets. Dataset A was used for training, and dataset B was used for external validation.

	Dataset A	Dataset B
Class 0	233	26
Class 1	339	53
Class 2	186	21
Total	758	100

**Table 2:** Quantitative results of the proposed model on dataset A. Fold 0, fold 1, fold 2, fold 3, and fold 4 are the respective results in the fivefold cross-validation experiment.

	Training Loss	Training Accuracy	Valid Loss	Valid Accuracy
Fold 0	0.0026	100%	0.0369	98.68%
Fold 1	0.0026	100%	0.1295	96.69%
Fold 2	0.0044	100%	0.0560	98.01%
Fold 3	0.0042	100%	0.0423	98.68%
Fold 4	0.0017	100%	0.0401	98.00%
Average	0.0031	100%	0.0618	98.15%

**Table 3:** Accuracy, sensitivity, specificity, PPV, and NPV of the recognition of low-risk high myopia (class 1).

	Accuracy	Sensitivity	Specificity	PPV	NPV
Human	93.00%	94.34%	91.48%	92.59%	93.48%
DCNNs	99.00%	100.00%	97.87%	98.14%	100.00%

**Table 4:** Accuracy, sensitivity, specificity, PPV, and NPV of the recognition of high-risk high myopia (class 2).

	Accuracy	Sensitivity	Specificity	PPV	NPV
Human	98.00%	95.24%	98.73%	95.23%	98.73%
DCNNs	99.00%	95.24%	100.00%	100.00%	98.75%

**Table 5:** Overall performance of the multiclassification task.

	Kappa Score	Jaccard Score
Human	0.8842	0.8693
DCNNs	0.9834	0.9801

## Data Availability

The datasets analyzed during the current study are available from the corresponding author upon reasonable request.
